# Data on mitochondrial ultrastructure of photoreceptors in pig, rabbit, and mouse retinas

**DOI:** 10.1016/j.dib.2020.105544

**Published:** 2020-04-20

**Authors:** Taku Ozaki, Shinto Utsumi, Takeshi Iwamoto, Makoto Tanaka, Hiroshi Tomita, Eriko Sugano, Eri Ishiyama, Kinji Ishida

**Affiliations:** a Department of Biological Science, Graduate School of Science and Engineering, Iwate University, 4-3-5 Ueda, Morioka, Iwate 020-8551, Japan; b Technical Support Center for Life Science Research, Iwate Medical University, Morioka, Japan

**Keywords:** Retina, Photoreceptor, Rod, Cone, Inner segment, Ellipsoid, Mitochondria, SEM imaging, CIS, cone photoreceptor inner segment, RIS, rod photoreceptor inner segment, SEM, scanning electron microscopy, OS, outer segment, IS, inner segment, ONL, outer nuclear layer, OPL, outer plexiform layer, INL, inner nuclear layer, IPL, inner plexiform layer, GCL, ganglion cell layer

## Abstract

Photoreceptors are one of the most energy-consuming cell types within the human body. To meet their high energy demand, photoreceptors possess a mitochondrial cluster in the inner segment of the cell. Interestingly, in several species, the inner segment of cone photoreceptors contains extremely large mitochondria that exceed 2 µm in diameter, called mega-mitochondria. We previously reported that pig retinas also contain mega-mitochondria, however, there are few reports whether mega-mitochondria are present in mammalian photoreceptors. In the present experiment, we analyzed pig, rabbit, and mouse photoreceptors under a scanning electron microscope (SEM), and compared the mitochondrial morphology. Our data showed that all three species present numerous mitochondrial clusters in the ellipsoid zone of photoreceptors, adjacent to the outer segment. In the pig retina, the inner segments of cone and rod photoreceptors were localized in different layers; consequently, we were able to distinguish them easily. Mega-mitochondria were identified only in the inner segment of cone photoreceptors. Also, mitochondria of cone photoreceptors, including mega-mitochondria, were dense cristae and high electron-densities compared to those of rod photoreceptors. In the rabbit retina, cone photoreceptors were existed within the layer of rod photoreceptor outer segment. The rod photoreceptors had a characteristic long outer segment. Cone photoreceptors had a short outer segment, and also had a thick inner segment compared to rod photoreceptors. Most of the mitochondria present in the rod photoreceptor inner segment were long and narrow, whereas mitochondria of cone photoreceptors were fragmented and short. Mega-mitochondria was not detected in rabbit retina. In the mouse retina, most of the photoreceptor cells were rod photoreceptors. Since the shape of the inner segments were very similar, we distinguished cone and rod photoreceptors based on the shape of the outer segments. Some mitochondria of both rod and cone photoreceptors were long and narrow, and there was no significant difference in mitochondrial morphology. Our data showed that mitochondrial morphology in the inner segment of photoreceptors vary among mammalian species. Although mega-mitochondria were present in pig photoreceptors, we could not observe their presence in rabbit nor mouse retinas. To our knowledge, this is a first experiment that perform the wide field observation of rabbit and mouse retina using electron microscopy, and that compare the mitochondrial morphology of photoreceptor cells in pig, rabbit and mouse.

Specifications table

**Table:** 

Subject	Cell Biology
Specific subject area	Mitochondrial ultrastructure of the retinas
Type of data	Images
How data were acquired	Scanning electron microscopy (SEM)
Data format	Raw and processed
Parameters for data collection	Porcine, rabbit, and mouse retinas were isolated from each eye bolls. We have made sections from each retina and observed mitochondrial morphology.
Description of data collection	Each retina are fixed and embedded in Epon 812. Ultrathin sections were cut with an ultramicrotome and stained with 1% uranyl acetate and lead citrate. Then the samples were observed under a SEM.
Data source location	Department of Biological Science, Graduate School of Science and Engineering, Iwate University, Morioka, Japan and Technical Support Center for Life Science Research, Iwate Medical University, Morioka, Japan
Data accessibility	Mendeley
	Direct URL to data: https://data.mendeley.com/datasets/46rcktkpyc/1
Related research article	T. Iwamoto, E. Ishiyama, K. Ishida, T. Yamashita, H. Tomita, T. Ozaki, Presence of calpain-5 in mitochondria, Biochem. Biophys. Res. Commun. 504 (2018) 454–459, doi:10.1016/j.bbrc.2018.08.144. [1]

## Value of the data

• To our knowledge, this is the first observation on a wide-field, high-resolution analysis of rabbit and mouse retinas using a unique scanning electron microscopy (SEM).
• Few reports have observed mitochondrial morphology in the retina, and this experiment may be useful to retinal researchers.
• The SEM technique can provide high-resolution images with a wide field view and is substantially useful in the field of morphology research.
• Our data showed that mega-mitochondria are not present in rabbit and mouse photoreceptors. Currently, there is little understanding of retinal mega-mitochondria. Therefore, the present data could be valuable for researchers.
• Mitochondrial dysfunction has been implicated in neurodegenerative diseases [2]. Thus, our data on mitochondrial morphology of retinal photoreceptors would be valuable to not only morphology research but also several fields of clinical research.

## 1 Data description

**Fig. 1:**
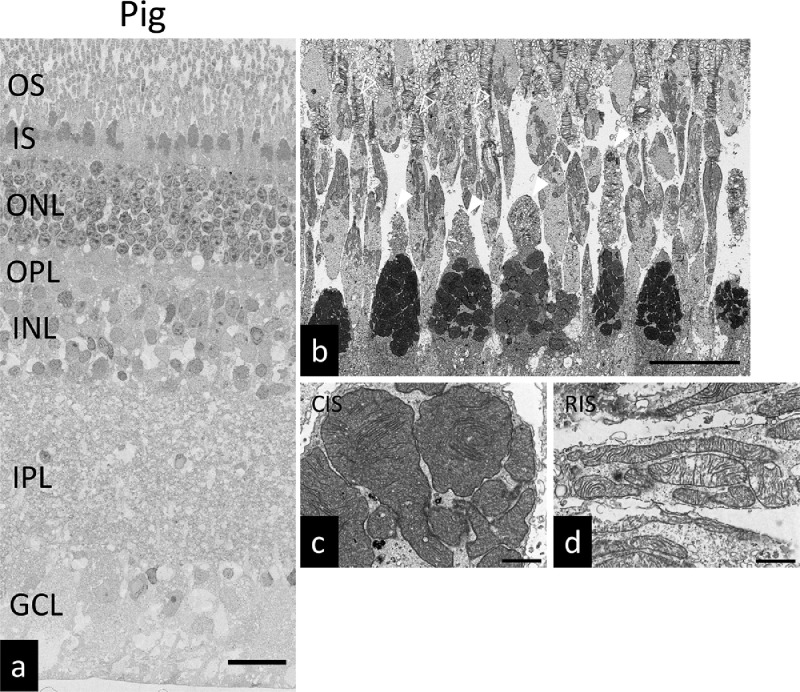
Ultrastructure of the porcine retina. (a) SEM micrograph of the porcine retina. Scale bar: 25 µm. (b) SEM micrograph of the porcine photoreceptors. White arrow heads: cone photoreceptors, Arrow heads: rod photoreceptors. Scale bar: 10 µm. (c) Magnified view of cone photoreceptor inner segment. Scale bar: 1 µm. (d) Magnified view of rod photoreceptor inner segment. Scale bar: 1 µm. OS: outer segment, IS: inner segment, ONL: outer nuclear layer, OPL: outer plexiform layer, INL: inner nuclear layer, IPL: inner plexiform layer, GCL: ganglion cell layer. CIS: cone photoreceptor inner segment. RIS: rod photoreceptor inner segment.

**Fig. 2:**
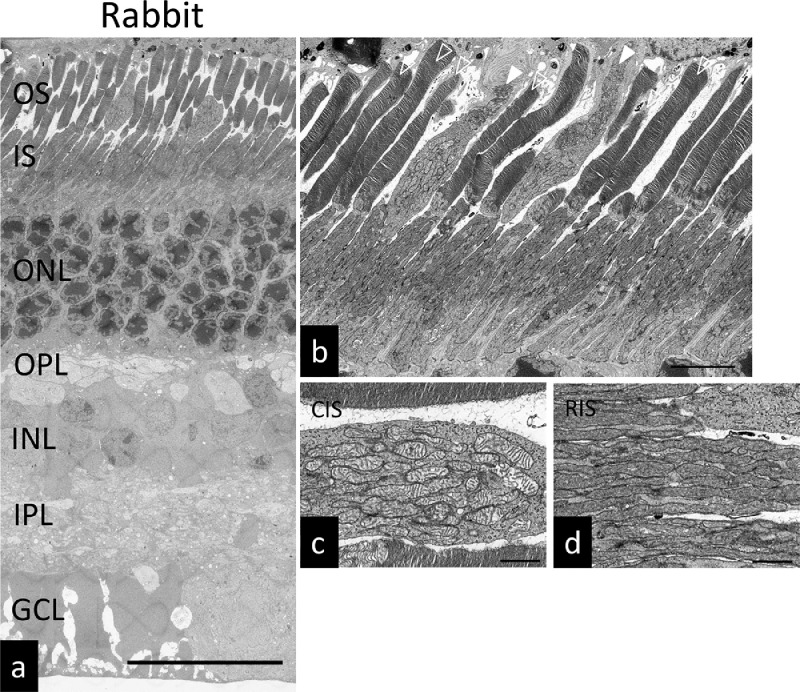
Ultrastructure of the rabbit retina. (a) SEM micrograph of the rabbit retina. Scale bars: 25 µm. (b) SEM micrograph of the rabbit photoreceptors. White arrow heads: cone photoreceptors, Arrow heads: rod photoreceptors. Scale bar: 5 µm. (c) Magnified view of cone photoreceptor inner segment. Scale bar: 1 µm. (d) Magnified view of rod photoreceptor inner segment. Scale bar: 1 µm. OS: outer segment, IS: inner segment, ONL: outer nuclear layer, OPL: outer plexiform layer, INL: inner nuclear layer, IPL: inner plexiform layer, GCL: ganglion cell layer. CIS: cone photoreceptor inner segment. RIS: rod photoreceptor inner segment.

**Fig. 3:**
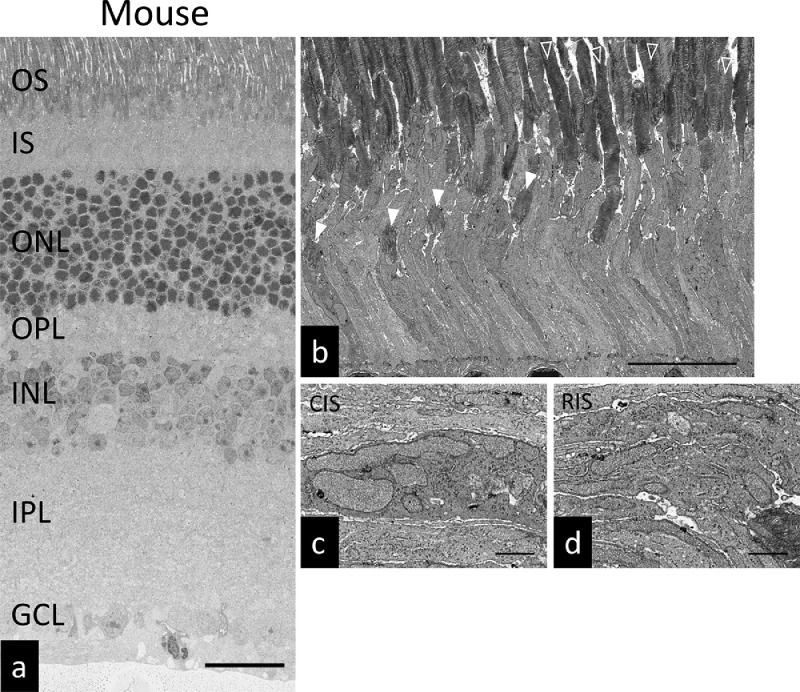
Ultrastructure of the mouse retina. (a) SEM micrograph of the mouse retina. Scale bar: 25 µm. (b) SEM micrograph of the mouse photoreceptors. White arrow heads: cone photoreceptors, Arrow heads: rod photoreceptors. Scale bar: 10 µm. (c) Magnified view of cone photoreceptor inner segment. Scale bar: 1 µm. (d) Magnified view of rod photoreceptor inner segment. Scale bar: 1 µm. OS: outer segment, IS: inner segment, ONL: outer nuclear layer, OPL: outer plexiform layer, INL: inner nuclear layer, IPL: inner plexiform layer, GCL: ganglion cell layer. CIS: cone photoreceptor inner segment. RIS: rod photoreceptor inner segment.

Our raw data files are uploaded in the Mendeley Data (https://data.mendeley.com/datasets/46rcktkpyc/1). We previously reported that pig retinas contain mega-mitochondria [1]. As there are few reports of mega-mitochondria present in mammalian photoreceptor cells, we evaluated retinal morphology in the pig (Fig. 1), rabbit (Fig. 2), and mouse (Fig. 3) using a scanning electron microscopy (SEM). To obtain high-resolution images with a wide field of view, we used a unique SEM method [3]. In conventional SEM, embedding and sectioning steps are not required, and only the surfaces of specimens are viewed. In contrast, our SEM technique involves mounting single or serial ultrathin sections on a glass slide, staining with heavy metals, and scanning backscattered electrons. By digitally “stitching” together contiguous SEM images, a wide field image can be obtained at high resolution. Fig. 1a showed electron microscope images of porcine retina. Fig. 1b showed electron microscope images of porcine photoreceptor cell layer. Fig. 1c showed the magnified view of cone inner segment in Fig. 1a. Fig. 1d showed the magnified view of rod inner segment in Fig. 1a. Fig. 2a showed electron microscope images of rabbit retina. Fig. 2b showed electron microscope images of rabbit photoreceptor cell layer. Fig. 2c showed the magnified view of cone inner segment in Fig. 2a. Fig. 2d showed the magnified view of rod inner segment in Fig. 2a. Fig. 3a showed electron microscope images of mouse retina. Fig. 3b showed electron microscope images of mouse photoreceptor cell layer. Fig. 3c showed the magnified view of cone inner segment in Fig. 3a. Fig. 3d showed the magnified view of rod inner segment in Fig. 3a.

## 2 Experimental design, materials, and methods

### 2.1 Experimental design

The retina plays pivotal roles in enabling vision, as it is involved in light stimuli reception and signal transmission to the visual cortex. The vertebrate retina comprises several cell layers including ganglion, bipolar, amacrine, horizontal, and photoreceptor cells [4]. Moreover, photoreceptors exhibit functional specialization; rod photoreceptor cells mediate scotopic vision, while cone photoreceptor cells mediate photopic vision [4,5]. Light capture and conversion into electric signals (phototransduction), as well as signal transmission from photoreceptors to subsequent retinal neurons require vast amounts of energy [6,7]. To supply this high-energy demand, numerous mitochondria are located in the inner segment of photoreceptors.

In some species such as killifish, shrews, and zebrafish the inner segment of cone photoreceptors contains heterogeneous mitochondria that are over 2 µm in diameter, known as mega-mitochondria or giant mitochondria [8], [9], [10]. It is generally accepted that cone photoreceptors consume more energy than rod photoreceptor cells [11], mega-mitochondria is considered as responsible for the high energy production required in these cells [10]. However, the physiological significance of these characteristic mitochondria is still not fully understood. In our previous study, we have shown that pig retinas also contain mega-mitochondria, which are similar to the ones observed in zebrafish in that localized only in the inner segment of cone photoreceptors and exist densely with each other [1]. However, few reports of retinal mega-mitochondria in other mammals exist. Further investigation of other mammal species is needed to elucidate the biological significance of mega-mitochondria presence in photoreceptors. Therefore, we observed the pig, rabbit, and mouse retinas. To obtain high-resolution images with a wide field of view, we used a unique SEM method [3].

In the present experiment, we investigated whether mega-mitochondria are present in the inner segment of photoreceptors from rabbit and mouse retina, using electron microscopy techniques. We also compared the morphology of mitochondria present in the inner segment of photoreceptors from pig, rabbit and mouse retinas.

### 2.2 Animals

All experiments were carried out in accordance with the U.K. Animals (Scientific Procedures) Act, 1986 and associated guidelines (EU Directive 2010/63/EU for animal experiments). The protocol was approved by the Committee for the Use of Live Animals at the Iwate University. Pig eyes were obtained from a slaughterhouse (Iwachiku, Iwate, Japan). New Zealand White (NZW) rabbits and C57BL/6 mice were obtained from Japan SLC (Hamamatsu, Shizuoka, Japan). Eight-week-old rabbits were euthanized using isoflurane, and their eyes were enucleated for retinal dissection. Eight-week-old mice were euthanized by isoflurane inhalation and perfused by fixation solution; their eyes were also enucleated for retinal dissection.

### 2.3 SEM imaging using backscattered electrons

We performed SEM to evaluate retinal morphology. In conventional SEM, embedding and sectioning steps dose not required and specimens have been observed only the surfaces. The SEM technique used in the present experiment involves mounting single or serial ultrathin sections on a glass slide, staining them with heavy metals and scanning backscattered electrons. By digitally “stitching” together contiguous SEM images, a wide field image can be obtained at high resolution. All procedures were conducted as described by Kataoka et al. [3]. The anterior structures, such as the cornea, lens, and iris, were removed and stored overnight at 4 °C in a fixative solution (2.5% glutaraldehyde, 2% paraformaldehyde solution, 0.1 M sodium phosphate buffer, pH 7.4). The fixed eyes were rinsed twice in 0.1 M phosphate buffer (10 min/rinse), and the retinas were excised and sectioned into small pieces. The trimmed retinas were post-fixed with 1% osmium tetroxide for 2 h and then dehydrated with increasing concentrations of ethanol (50%, 70%, 80%, 90%, and 100%) for 15 min at each concentration. After dehydration, the samples were rinsed twice with QY-1 (15 min/rinse), once with a mixture of QY-1 and Epon 812 (TAAB Laboratory Equipment Ltd., Aldermaston, England) (1:1 ratio), and then embedded in Epon 812. Ultrathin sections (100 nm) were cut with an ultramicrotome (EM-UC6, Leica Microsystems, Wetzlar, Germany) and stained with 1% uranyl acetate and lead citrate. The samples were then observed under a scanning electron microscope (SU 8010, Hitachi, Tokyo, Japan). Regions of the ultrathin sections were imaged using backscattered electrons with an accelerating voltage of 1.5 kV, thereby enabling imaging at a high-resolution. After acquisition, contiguous SEM images were digitally stitched together using the image stitching plugin in Fiji/Image J software (https://imagej.net/Image_Stitching) to generate an entire retina at high resolution, sufficient to confirm the presence of mitochondrial morphology. The stitched SEM images were viewed with the virtual-slide software (NDP.view2).

## Appendix Supplementary materials

Image, application 1
